# Assessment of socio-relational self-efficacy in breast cancer patients: Italian validation of the social relationship coping efficacy scale (SRCE-I)

**DOI:** 10.1186/s40359-022-00966-7

**Published:** 2022-11-03

**Authors:** Samantha Serpentini, Giulia Silvestri, Antonio Catarinella, Grazia Cristaldi, Caterina Borgese, Giuseppe Deledda, Samuela Sommacal, Letizia Iannopollo, Vincenzo Calvo, Lucia Ronconi, Thomas V. Merluzzi

**Affiliations:** 1 Psychological Service of the Breast Cancer Unit, UOC Medical Oncology 2, UOSD Hospital Psychology, Veneto Institute of Oncology (IOV) – IRCCS, Via Gattamelata, 64, 35100 Padua, Italy; 2 Vita-Salute San Raffaele University, Milan, Italy; 3 Sacro Cuore Hospital- Don Calabria-IRCCS of Negrar (VR), Negrar, Italy; 4 Department of Philosophy, Sociology, Pedagogy and Applied Psychology (FISPPA), University of Padua, Padua, Italy; 5 Department of Psychology, University of Notre Dame, Notre Dame, IN USA

**Keywords:** Self-efficacy, Breast cancer, Social relationships, Social support, Coping efficacy, Quality of life, Validation study

## Abstract

**Background:**

Social relationship coping efficacy (SRCE) represents the ability to maintain or enhance social relationships in the context of serious illness. The purpose of the current study was to confirm the factor structure, psychometric properties, and utility of the Italian version of the SRCE scale.

**Methods:**

181 breast cancer patients completed the SRCE-Italian (SRCE-I), the Cancer Behavior Inventory–Brief/Italian (CBI–B/I), quality of life (QOL) measures (EORTC QLQ-C30; EORTC QLQ-BR23), and the Hospital Anxiety and Depression Scale (HADS).

**Results:**

The SRCE-I was internally consistent (Cronbach alpha = .95) and factor analysis confirmed that the SRCE-I was a unidimensional construct. In terms of validity, the SRCE-I was correlated with QOL (EORTC QLQ-30, Social Functioning, r = .33, Emotional Functioning, r = .57, and Global Health/Quality of Life; r = .54) and scales of the EORTC QLQ-BR23 (e.g., Future Perspective, r = .38; Breast Symptoms, −.31). SRCE-I was also correlated negatively with the HADS (r = −.72) and positively with the CBI–B/I (r = .79), a measure of coping efficacy (all ps < .001). Mediation analyses confirmed the utility of the SRCE-I scale as a mediating mechanism in enhancing social functioning and QOL.

**Conclusions:**

The SRCE-I is a structurally sound, reliable, and valid measure that assesses the ability to maintain or enhance social support and mitigate the loss of social support. The SRCE-I can be used as a screening measure to assess low efficacy for maintaining social support or as a measure to detect the change in efficacy for enhancing social support in interventions to improve the QOL of patients.

**Supplementary Information:**

The online version contains supplementary material available at 10.1186/s40359-022-00966-7.

## Background

Supportive personal relationships contribute substantively to the well‐being of cancer patients by fostering adjustment during cancer treatment and post-treatment [1, 2] as well as promoting positive quality of life [3–5], positive medical outcomes [6], and increased survival time [7, 8]. The importance of social support is evident from survey data of cancer patients’ perceptions of their needs for social support from family and friends [9] and intimate partners [10, 11]. Whereas patients are generally satisfied with the support they receive, that does not hold for all patients especially younger patients [12], who may be particularly vulnerable to anxiety in unsupportive relationships [13]. In contrast to the beneficial effects of social support, lack of social support can have negative personal effects [14], decrease QOL [15], and increase cancer recurrence and mortality [16, 17]. Thus, in the course of coping with serious illnesses such as cancer, maintaining or enhancing support may promote not only quality of life but also longevity.

A reduction of social support in the context of cancer is not uncommon and may be a function of the physical limitations that accompany the disease and its treatments [18–20]. In addition to the shrinking of social networks of cancer patients, there is also the issue of maintaining the quality of close personal relationships such as with a spouse or partner [10, 11, 21]. However, even with the best of intentions on the part of the support-giver, there may be detrimental effects of a mismatch between the need for support and the support provided [22]. This mismatch may take for form of not only neglecting to provide the support that was needed but also providing support when it is not needed, thereby undermining the patient’s personal agency concerning self-care and reinforcing “sick role” behavior [23]. Thus, in order to optimize social support and supportive relationships, the provider of support must assess the need and provide support accordingly [24]. However, the alignment of provision and need for support may not be the sole responsibility of the provider, but may also be a role that the support-recipient assumes [25]. That is, in contrast to the provider determining the conditions of the provision of support, the person with cancer can also play a role in shaping the matching of need with provision, which makes the support process bidirectional and dynamic as opposed to unidirectional and static. This last point is especially important for the current study because the model of persons with cancer being active agents of their well-being [26] and disclosing the diagnosis of cancer to patients by medical professionals are not as prevalent in Italy as in the US [27]. Thus, social support and its enactment may have some variations that are a function of the culture in which this process occurs. Therefore, it is important to study these processes in different cultures in order to document similarities and differences.

The focus on the patient’s role in the matching of need and provision of support was embodied in a new construct, social relationship coping efficacy (SRCE) that is grounded in Self-efficacy Theory [28, 29] and Self-regulation Theory, which assume that the individual has certain goals (e.g., increase intimacy with a partner) and engages in behaviors that may close the gap between a current state (e.g., loss of social support) and a desired goal state (e.g., regain, maintain, or enhance social relationships and social support) [30]. The SRCE scale [25] assesses the confidence of the person with cancer to execute behaviors such as “managing stress in my relationships”, “doing my part to help family members accept/ understand my diagnosis”, and “seeking emotional support from others.” As opposed to being a mere recipient of social support, SRCE emphasizes the agentic role of the cancer patient to create the conditions under which social support is optimized. Finally, SRCE might explain why some people are able to maintain or enhance social support despite the disease while others are not able to mitigate the loss of social support.

The psychometric properties of the 10-item SRCE scale include strong internal consistency and concurrent validity as well as unidimensionality. These properties have been replicated in a Greek version of the SRCE scale [31]. In addition, SRCE has been tested in a double mediation model [25] as a mediating mechanism between physical debilitation, which brings about loss of social support, and received social support. In that model, SRCE was a mediating mechanism that was positively related to social support, and thus, may account for recovery of social support that was compromised by physical debilitation. In addition, in the double mediation model, whereas SRCE was directly related to QOL outcomes (social/family well‐being and psychological distress) social support partially mediated the relationship between SRCE and those QOL outcomes [25]. These intricate relationships demonstrate the role of SRCE in potentially reversing negative outcomes due to the physical limitations that are endemic to cancer and its treatments. Thus, the SRCE scale may be useful in clinical practice to assess the patient’s agency for optimizing social support and in interventions that enhance social support and QOL.

In addition to the SRCE, a recently developed measure of social relationships, the Social Relational Quality Scale (SRQS) [32], assesses the current state of relationships along three dimensions: Family Intimacy, Family Commitment, and Friendships. This measure was also developed and used in the context of cancer and other serious illnesses and is correlated with hope [33] and acceptance of illness [34]. However, in contrast to the SRQS and other relationship assessment scales (e.g., Perceived Relationship Quality Components Inventory) [35], which are designed to be descriptive, the SRCE was designed not only for descriptive purposes but also for prediction. In the context of Self-Efficacy Theory, the SRCE scale is a measure of behavior expectancy for engaging in behaviors that might enhance personal relationships and social support in the near future. Thus, SRCE is an agentic, motivational construct that, in the context of Self-Regulation Theory [30], is tied to goals or outcomes, and therefore goes beyond descriptive utility.

The purpose of the current study was to examine the psychometric qualities including validity of the Italian version of the social relationship coping efficacy scale [25] on a sample of Italian women who were receiving treatment for breast cancer. In addition, the utility of SRCE as mediator between physical functioning and quality of life was examined. Given the high quality of the SRCE scale in a mixed sample of cancer patients in the USA [25] and in a sample of Greek breast cancer patients [31], the determination of its validity for Italian cancer patients is a logical extension of that prior work to provide evidence about its cross-cultural application. Therefore, the SRCE scale was translated into Italian and its reliability, structural validity, concurrent validity and utility in mediating the relationship between physical debilitation and quality of life outcomes were examined on a sample of Italian women with breast cancer. Based on prior work [25, 31], we hypothesized that the SRCE-Italian scale would be internally consistent, unidimensional in structure, and valid based on its relationships with a variety of measures that focused on social and emotional functioning as well as quality of life. Finally, mediation analyses were conducted to test the replication of SRCE as mediating mechanism between physical functioning and quality of life outcomes. In sum, we hypothesized that the SRCE construct would be culturally valid in Italy as it is in the US and Greece.

## Methods

### Participants

The inclusion criteria established for the present study were: breast cancer patient, at least 18 years old, having received or engaged in active cancer-related treatment (i.e., surgery, chemotherapy, radiation therapy, hormone therapy) and not in palliative care. Patients were receiving treatment at the Veneto Institute of Oncology-IRRCS (IOV) or the Sacro Cuore Hospital—Don Calabria-IRCCS in Negrar (VR). The exclusion criteria were: insufficient language skills to complete the questionnaires, deficits in functional autonomy, clinical conditions that hindered the completion of questionnaires independently such as severe psychiatric disorders, intellectual disability, or cognitive impairment (related to people, place, or time). Data were collected between May 31, 2016 and May 18, 2021.

Based on the inclusion and exclusion criteria, 181 patients participated in the study; 200 patients were initial approached and asked to participate, 19 declined resulting in 181 participants, a 90.5% participate rate. The age of the participants ranged from 27 to 75 years with a mean age of 50.44. Most of the participants (63%) were married/partnered, had a high school diploma (41%), were employed (69%), practiced religion (55%), and cohabitated with other people (90%). In terms of medical information, which was garnered from medical charts, at diagnosis, 35% were Stage I, 31% Stage II, 21% Stage III, 4% Stage IV, and 9% were not staged; 28% had metastatic disease. With regard to treatments, 80% had surgery, 44% had radiotherapy, 56% had chemotherapy, and 70% received hormone treatment. Table 1 contains more detailed information about the participants.

**Table 1:** Participant demographic and medical information (N = 181)

Variable	Mean (SD)/N (%)
**Age**	50.44 (9.99)
**Partnership status**	
Unmarried	22 (12%)
Married	114 (63%)
Live together	12 (7%)
Widowed	9 (5%)
Divorced	24 (13%)
**Educational level**	
Elementary school	3 (2%)
Middle school	39 (22%)
High school	74 (41%)
Graduation	65 (36%)
**Employment status**	
Worker	125 (69%)
Retired	17 (9%)
Homemaker	29 (16%)
Other	10 (6%)
**Religion/spirituality**	
Practicing religion	99 (55%)
Religious but not practicing	56 (31%)
Belief in something but no specific religious faith	12 (7%)
Atheist	8 (4%)
Agnostic	2 (1%)
Other	4 (2%)
**Living arrangements**	
Live alone	18 (10%)
Do not live alone	163 (90%)
**Family network (N = 102)**	
Partner	94 (92%)
Children	93 (91%)
Nephew	12 (12%)
Parents	20 (20%)
Grandparents	4 (4%)
Brothers/Sisters	23 (23%)
Other	6 (6%)
**Hobbies**	
No	26 (14%)
Yes	155 (86%)
**Volunteering**	
No	138 (76%)
Yes	43 (24%)
**Time since diagnosis**	
Less than 2 months	9 (5%)
Less than 6 months	22 (12%)
Less than 1 year	37 (20%)
Less than 2 year	41 (23%)
Less than 5 year	46 (25%)
More than 5 years	26 (14%)
**Tumor stage (N = 160)**	
I	56 (35%)
II	50 (31%)
III	34 (21%)
IV	6 (4%)
Not staged	14 (9%)
**Metastasis (N = 179)**	
Yes	50 (28%)
No	129 (72%)
**Chemotherapy purpose (N = 146)**	
Curative	145 (99%)
Palliative	1 (1%)
**Toxicity**^**1**^** of Treatments(N = 150)**	
1–2	61 (41%)
3–4	89 (59%)
**Surgery (N = 179)**	
Yes	143 (80%)
No	35 (19%)
Pending	1 (1%)
**Radiotherapy (N = 179)**	
Yes	79 (44%)
No	94 (53%)
Ongoing	6 (3%)
**Chemotherapy (N = 180)**	
Yes	100 (56%)
No	40 (22%)
Ongoing	40 (22%)
**Hormone treatment (N = 179)**	
Yes	126 (70%)
No	53 (30%)

^1^The following refer to grades of toxicity: 1 = Highly toxic and Severely irritating, 2 = Moderately toxic and Moderately irritating, 3 = Slightly toxic and Slightly irritating, 4 = Practically non-toxic and not an irritant

### Procedure

In order to broaden the sample, the Veneto Institute of Oncology-IRRCS (IOV) created a partnership with the Sacro Cuore Hospital—Don Calabria-IRCCS in Negrar (VR) to have a more geographically diverse sample of Italian women with breast cancer. Patients who met the criteria for participation were given a survey packet with a consent form and measures. The consent form was prepared according to the procedures established by current laws, regulations, and ethical standards for the treatment of human research participants. The study was approved by the IOV—IRCCS Research Ethics Committee, all procedures were in accordance with the 1964 Helsinki declaration and its later amendments, and all participants provided informed consent.

For those who consented to participate, the measures packet was completed in one session, which took at about 45 min. Participants were encouraged to complete all items to the best of their ability. In order to maintain confidentiality, a code was attached to the packet, which was separated from the consent forms. All consent forms were kept in the locked files of the researchers at each location.

### Measures

The participants completed a packet containing: the informed consent form, in which the study’s objectives were presented, a release of information form that allowed the researchers to access specific medical information from the patients’ medical records, a personal data sheet with items to gather socio-demographic and self-report medical information, and five self-report surveys described below.

#### Personal datasheet

The participants completed multiple-choice and open-ended questions related to their socio-demographic information including age, marital status, level of education, occupation, spirituality, family network, hobbies, and volunteering. Other information about time since diagnosis, tumor stage, metastasis, and therapies was derived from the patients’ medical records.

#### Social relationship coping efficacy (SRCE-I)

The social relationship coping efficacy scale [25] is a 10-item scale that assesses self-efficacy for the ability to engage in behaviors that might foster maintenance or enhancement of close social relationships in the context of illness. Based on the English version, the SRCE-Italian consists of 10 items (e.g., managing stress in my relationships, adjusting to the ways cancer affects my family) that are rated on a Likert-type scale ranging from 1 (not at all confident) to 9 (totally confident) with respect to performing the behavior. Cronbach's alphas for the English [25] and Greek [31] versions (0.97 and 0.87, respectively) were very strong and validity was confirmed with significant correlations with multiple measures of social functioning as well as social, emotional, and functional well-being [25]. Factor analyses confirmed that the English and Greek versions of the SRCE were unidimensional.

##### Translation of the SRCE-I

The translation of the SRCE-I was performed in accordance with the EORTC guidelines [36], which began with obtaining permission from the original authors [25] to translate the SRCE into Italian. The initial translation from English was completed independently by two native speakers of Italian with excellent English skills, who were employed at the Veneto Institute of Oncology. The two Italian versions were compared and, based on consensus, a third version was compiled, which was back-translated to English by a native English speaker with excellent fluency in Italian. That person compared the back-translated version with the original English version and resolved differences in wording. Taking into account the back-translated English version, the original version, and the Italian version, the initial Italian translators collaborated on a further revision that resolved differences and provided a consensus translation of the SRCE into Italian (SRCE-I). A pilot phase was subsequently conducted, which included: (1) an administration of the SRCE-I to five patients who were representative of the sample included in this study; (2) a thorough interview to probe the patients’ understanding of the items; (3) the collection and recording of any perceived difficulties with items and suggestions, and (4) an analysis of the SRCE-I qualitative data. The final version of the SRCE-I was back-translated into English and sent to the original authors, who evaluated and approved that version.

#### Hospital anxiety and depression scale (HADS)

The Hospital Anxiety and Depression Scale (HADS) [37, 38] assesses the experience of anxiety (7 items) and depression (7 items) during the previous week. Items are rated on a 4-point Likert-type scale from 0 to 3 that varies with items but generally assesses the frequency of the experience. Scoring consists of summing the items within each of the anxiety and depression scales and summing those components to form a total score. Validity [38] and utility of the HADS has have been established in many cultural contexts [39]. Cronbach’s alpha internal consistency coefficients for the HADS (Italian) in the current sample were the following: HADS-A = 0.87, HADS-D = 0.83, HADS-Total = 0.91.

#### Cancer behavior inventory—brief Italian version (CBI–B/I)

The CBI–B/I [40] is a 12-item measure of self-efficacy for coping with cancer. Whereas there is a factor structure to the CBI–B/I [40] and the original English version [41], it is generally used as a unidimensional scale. The internal consistency reliability of the CBI–B/I is strong (alpha = 0.86) and validity has been established by significant correlations with the EORTC QLQ-C30 [42], Mini-MAC [43], and HADS [37]. In addition, differences in CBI–B/I scores comparing high versus low levels of the ECOG-Performance Status measure [44] supported the clinical utility of the CBI–B/I. Cronbach’s alpha value of internal consistency for the CBI–B/I total score in the current sample was 0.93.

#### EORTC QLQ-C30

The EORTC QLQ-C30 [42] is a quality-of-life measure with solid psychometric properties. This measure has been translated and validated in Italian [45, 46]. The EORTC QLQ-C30 consists of 30 items organized into five areas of functioning (physical, role, emotional, cognitive, and social), nine symptom subscales/items (fatigue, nausea/vomiting, pain, dyspnea, insomnia, appetite loss, constipation, diarrhea, and financial difficulties) and a Global Health/Quality of Life subscale. Items are rated on a 4-point Likert-type scale, ranging from 1 (not at all) to 4 (very much), except for the two items related to the Global Health/Quality of Life subscale that are rated on a 7-point scale, ranging from 1 (very poor) to 7 (excellent). Mean scale scores are converted to a 0–100 scale using the linear transformation in the EORTC manual. Higher scores on the symptom scales indicate a greater degree of symptom burden, whereas higher scores on the functioning and Global Health/Quality of Life subscales indicate better functioning and better quality of life.

#### EORTC QLQ BR23

The EORTC QLQ BR23 [47] is a breast-cancer-specific quality of life measure that was designed to accompany the EORTC QLQ C-30. The BR-23 contains 23 items that map onto 6 scales: sexual functioning, sexual enjoyment, future perspective, systemic therapy side effects, breast-related symptoms/arm-related symptoms. Items are rated on a Likert scale (1 = no, 2 = a little, 3 = a lot, 4 = very much) and lower scores indicate a more positive perception of quality of life.

### Data analysis plan

Statistical analyses were conducted with Statistical Package for the Social Sciences (IBM SPSS Statistics, Version 27, IBM Corp., Armonk, N.Y., USA). Reliability was tested to determine the internal consistency of the social relationship coping efficacy scale—Italian (SRCE-I) using Cronbach’s alpha coefficient. Structural validity was evaluated using principal axis factor analysis. Concurrent validity was assessed through correlations of the SRCE-I with other measures: HADS, CBI–B/I, EORT QLQ-30, and EORTC QLQ-BR23. The normality for the distributions of all measures was assessed with the Shapiro–Wilk test. Significant *p*-values (*p* < 0.001) were found for all variables therefore, non-parametric Spearman rho coefficients were used to compute the relationship of the SRCE-I with other measures (Table 3). Bonferroni adjustment for multiple correlations was used based on the maximum number of scales in the criterion measure (i.e., the 10 Physical Symptom Scales in EORTC QLQ-30).

The relationship of each demographic and medical variable with SRCE-I was evaluated initially at the univariate level using simple regression analysis followed by the multivariate level with multiple regression model. In addition, mean SRCE differences were tested between married/partnered participants and those who were not as well as between those who were in Stages I and II versus Stages III and IV at diagnosis. Age differences that emerged were investigated with regression analyses and measurement invariance. Multigroup Confirmatory Factor Analyses (MCFA) were analyzed using the lavaan package [48] in R software with the estimator of diagonally weighted least squares (DWLS) because such an estimator is suitable for Likert-type scales used with the SRCE-I.

Finally, as a test of the utility of the SRCE-I to potentially mitigate the loss of social support, mediation analyses were conducted with SRCE-I as a mediator between a measure of physical functioning (EORTC QLQ-30 Physical Functioning) and three dependent measures: Social Functioning and Global Health/Quality of Life (EORTC QLQ-30) and the HADS total score (Anxiety and Depression). Mediation analyses were conducted using Hayes’ PROCESS [49] program embedded in SPSS Statistics 28.0. Confidence interval for each effect was estimated by bootstrap (95% confidence, 5000 bootstrap samples).

## Results

### Reliability analysis

Cronbach’s alpha for the SRCE-I 10 item-scale was 0.95 indicating very high internal consistency reliability. All items were important for inclusion based on the item-to-total score correlations for each item, which ranged from 0.71 to 0.83 (Table 2). In addition, a consistently high Cronbach’s alpha (alpha = 0.94) was obtained even when one item at a time was deleted and alpha was recomputed. Collectively, these data indicate strong internal consistency and substantial inter-relationship among the items in the SRCE-I.

**Table 2:** Item statistics, factor loadings, and communality values for each item of the SRCE-I

Item	Mean	SD	Skewness	Kurtosis	Item-total correlation	Factor loading	Communality
1. Doing my part to maintain close relationships	6.99	1.85	−0.88	0.14	0.71	0.72	0.52
2. Managing stress in my relationships	6.16	1.94	−0.48	−0.36	0.75	0.77	0.59
3. Asking for help when I need it	6.65	2.07	−0.82	−0.04	0.77	0.79	0.63
4. Seeking emotional support from others	6.34	2.16	−0.72	−0.24	0.77	0.80	0.63
5. Coping with stress in my close relationships	5.84	2.21	−0.41	−0.62	0.83	0.86	0.74
6. Doing my part to help family members accept/ understand my diagnosis	6.73	1.95	−0.86	0.30	0.78	0.80	0.64
7. Doing my part to help my friends accept/understand my diagnosis	6.59	2.08	−0.93	0.42	0.81	0.83	0.69
8. Adjusting to the ways that cancer affects my family	6.28	1.94	−0.74	0.32	0.77	0.80	0.64
9. Coping with the ways that cancer affects my personal relationships	6.33	1.94	−0.66	0.08	0.82	0.85	0.73
10. Managing conflict with those closest to me	6.05	2.17	−0.51	−0.44	0.76	0.78	0.61

### Structural analysis

A significant Bartlett’s Test of Sphericity (Chi-Square = 1590.39, *p* < 0.001) indicated statistically strong correlations among the items, rejecting the null hypothesis that the SRCE-I items were independent of each other. In addition, a Kaiser–Meyer–Olkin Measure (KMO) Index of 0.897 confirmed the adequacy of sampling. Exploratory factor analysis, using the principal axis factor (PAF) method, extracted a single factor with an eigenvalue greater than 1, which confirmed the factor structures in the English [25] and Greek versions of the SRCE scale [31]. In addition, the one-factor model explained 68% of the total variance. All factor loadings were greater than 0.70 and all communalities exceeded 0.50, indicating all variables contributed to the solution (Table 2).

### Validity analyses

The concurrent validity of the SRCE-I was confirmed by significant correlations with other measures (Table 3). In particular, the SRCE-I was positively correlated with the CBI–B/I total score (r = 0.79, *p* < 0.05), which indicated a positive correlation with self-efficacy for coping with cancer. Importantly for concurrent validity with similar constructs, the SRCE-I was significantly positively correlated with the EORTC QLQ-30 Role (r = 0.30, *p* < 0.05) and Social Functioning scales (r = 0.33, *p* < 0.05), indicating that efficacy for maintaining or enhancing social relationships is positively related to social adjustment. Furthermore, positive relationships with the Global Health/Quality of Life (r = 0.54, *p* < 0.05), and Emotional Functioning (r = 0.57, *p* < 0.05, scales of the EORT QLQ-30 as well as inverse correlations with the Anxiety (r = −0.64, *p* < 0.05) and Depression (r = −0.69, *p* < 0.05) scales of the HADS supported the relationship between maintaining or enhancing social support (i.e., the SRCE-I) and more general psychosocial functioning. Finally, the significant relationships and the direction of the correlations between the SRCE-I and the physical, functional, and symptom scales of the EORTC QLQ-30 and EORTC QLQ-BR23, with few exceptions, supported the relationship between maintaining or enhancing close personal relationships and physical functioning.

**Table 3:** Correlation SRCE with others measures: concurrent validity coefficients

Measure	Scale	Correlation with SRCE-I
**EORTC QLQ-30**	**Quality of Life Scales**	
	Global health: Quality of life Role functioning	.54* .30*
	Social functioning	.33*
	Emotional functioning	.57*
	Cognitive functioning	.37*
	Physical functioning	.27*
	**Physical Symptom Scales**	
	Pain	−.31*
	Fatigue	−.34*
	Insomnia	−.35*
	Nausea and vomiting	−.27*
	Dyspnea	−.21*
	Appetite Loss	−.21*
	Constipation	−.15
	Diarrhea	−.22*
	Financial difficulties	−.23*
**EORTC QLQ-BR23**	**Scales**	
	Sexual functioning	.29*
	Future perspective	.38*
	Systemic therapy side effects	-.31*
	Breast symptoms	-.31*
	Arm symptoms	-.15
**CBI–B/I**	Total CBI–B/I Score	.79*
**HADS**	**Scales**	
	Anxiety	-.64*
	Depression	-.69*
	Total HADS Score	-.72*

Sexual enjoyment scale is absent because not enough participants completed the scale to report results

***p < .001; **p < .01; *p < .05

*p < .05 (Bonferroni adjusted for multiple comparisons; alpha corrected for each correlation = alpha global/maximum number of scales in the same measure = .05/10 = 0.005)

### SRCE-I and demographic and medical variables

A multivariate, multiple regression analysis with all demographic and medical variables included revealed that only age (b = −0.34, *p* = 0.003) was a statistically significant predictor of SRCE-I. In addition, the mean of SRCE-I for those who had a partner (Married, Living together) and for those who did not (Unmarried, Widowed, Divorced) was similar (M = 64.74 SD = 17.28 and M = 62.20 SD = 15.34, respectively) and no difference between the two groups was found (t = 0.94 df = 179 *p* = 0.349). Finally, the mean SRCE-I for those who were in Stage I and Stage II compared to those who were in Stage III or Stage IV at diagnosis was similar (M = 62.47 SD = 18.13 and M = 66.72 SD = 14.12, respectively) and no statistically significant difference was found (t = −1.34 df = 144 *p* = 0.183).

#### Multigroup invariance analysis for younger versus older participants

As a follow up to the finding that the SCRE-I was related to age, a multigroup CFA was used to test measurement invariance across two age groups (< 50 years old, n = 102 versus > 50 years old, n = 79). Five models were constructed in the multigroup CFA; a configural model (Model 1: a one-factor structure of SRCE-I), a metric invariance model (Model 2: a model that constrained all factor loadings to be equal between age groups), a scalar invariance model (Model 3: a model that constrained all factor loadings and item intercepts to be equal across age groups), a factor variance invariance model (Model 4: a model that constrained all factor loadings, all item intercepts and factor variance to be equal across age groups) and a factor mean invariance model (Model 5: a model that constrained all factor loadings, all item intercepts, factor variance and factor means to be equal across age groups). The five models were then mutually compared to assess measurement invariance: fit indices (i.e., Chi-square, CFI, RMSEA, and SRMR) of Model 1 were compared with those of Model 2 for metric invariance; fit indices of Model 2 were compared with those of Model 3 for scalar invariance; fit indices of Model 3 were compared with those of Model 4 for factor variance invariance; and fit indices of Model 4 were compared with those of Model 5 for factor mean invariance. Measurement invariance was supported when no significant Delta Chi-squares, Delta CFI >  − 0.01, Delta RMSEA < 0.015, and Delta SRMR < 0.01 occurred in the model comparisons [50] between the age groups.

The configural invariance model showed good fit indices (Chi-square = 104.94, df = 62, *p* = 0.001, CFI = 0.998, RMSEA = 0.088, SRMR = 0.050) and measurement invariance was supported by comparison between models up through Model 4 (factor variance invariance). In fact, only the comparison between Model 4 and Model 5 (factor mean invariance) showed significant differences in fit indices: Delta Chi-square = 173.77, df = 1, *p* < 0.001, Delta CFI = −0.01, Delta RMSEA = 0.07, Delta SRMR = 0.00). These results indicated the presence of the same factor structure of SRCE-I, with the same factor loadings, the same intercepts and the same factor variance, in both age groups. Only factor means were different in the two groups with a higher level in the younger age group (M = 67.06 vs M = 59.97) and this was expected based on the regression results.

### Utility of the SRCE-I as a mediator

**Fig. 1:**
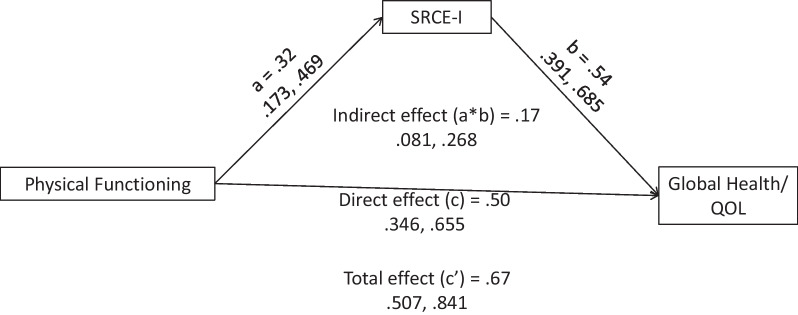
Mediation of the relationship between physical functioning (EORTC-QLQ-30) and Global Health Status: Quality of Life (EORTC-QLQ-30) by social relationship coping efficacy—Italian

**Fig. 2:**
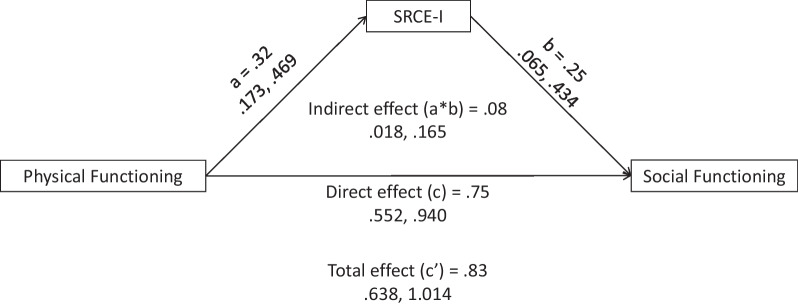
Mediation of the relationship between physical functioning (EORTC-QLQ-30) and Social Functioning (EORTC-QLQ-30) by social relationship coping efficacy—Italian

**Fig. 3:**
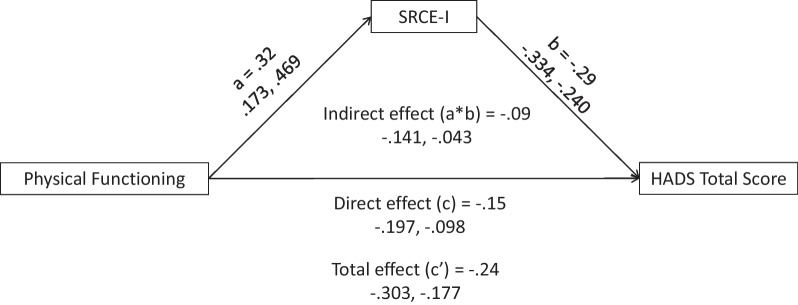
Mediation of the relationship between physical functioning (EORTC-QLQ-30) and the Hospital Anxiety and Depression Scale (Total Score) by social relationship coping efficacy—Italian

The loss of social support [5, 6] may be due, in part, to decrements in physical functioning that many times accompany cancer and its treatments. Based on prior research [25] that investigated SRCE as a mechanism that may mitigate social support losses, analyses were conducted as a test of SRCE-I as a mediator between Physical Functioning (EORTC-QLQ-30) and Global Health/Quality of Life (Fig. 1; EORTC QLQ-30), Social Functioning (Fig. 2; EORTC QLQ-30), and depression/anxiety (Fig. 3; HADS total score). Based on the significance of the indirect effects (Table 4), in these analyses, the SRCE-I was a significant mediator between the Physical Functioning scale of the EORTC QLQ-30 and the three dependent measures: Social Functioning (Indirect Effects Est. = 0.08, 95% CI: 0.018, 0.165); Global Health/Quality of Life (Indirect Effects Est. = 0.17, 95% CI: 0.081, 0.268); and HADS: Depression/Anxiety (Indirect Effects Est. = −0.09, 95% CI: −0.141, −0.043).

**Table 4:** Direct, indirect, and total effects of the mediation model

Outcome	Effect	Path	Estimate	95% CI	
				Lower	Upper
Global Health/Quality of Life	Direct effect	Physical Functioning → SRCE-I	0.32	0.17	0.47
		SRCE-I → Global QOL	0.54	0.39	0.69
		Physical Functioning → Global QOL	0.50	0.35	0.66
	Indirect effect	Physical Functioning → SRCE-I → Global QOL	0.17	0.08	0.27
	Total effect	Physical Functioning → Global QOL	0.67	0.51	0.84
Social Functioning	Direct effect	Physical Functioning → SRCE-I	0.32	0.17	0.47
		SRCE-I → Social Functioning	0.25	0.07	0.43
		Physical Functioning → Social Functioning	0.75	0.55	0.94
	Indirect effect	Physical Functioning → SRCE-I → Social Functioning	0.08	0.02	0.17
	Total effect	Physical Functioning → Social Functioning	0.83	0.64	1.01
HADS total score	Direct effect	Physical Functioning → SRCE-I	0.32	0.17	0.47
		SRCE-I → HADS Total Score	−0.29	−0.33	−0.24
		Physical Functioning → HADS Total Score	−0.15	−0.20	−0.10
	Indirect effect	Physical Functioning → SRCE-I → HADS Total Score	−0.09	−0.14	−0.04
	Total effect	Physical Functioning → HADS Total Score	−0.24	−0.30	−0.18

Confidence interval for indirect effect was estimated with bootstrap method (95% confidence, 5000 bootstrap samples). CI = confidence interval

## Discussion

These results support the conclusion that the SRCE-I is a reliable, unidimensional, and valid measure, which replicates findings from the original measure [25] and also a recent Greek translation and validation of the measure [31]. Taken together, data from these three studies indicate that the SRCE scale embodies a robust construct that has relevance in a variety of cultural settings. Furthermore, the mediation analyses with the SRCE-I replicated critical portions of the mediation models involving the original SRCE measure in that the SRCE-I mediated the relationship between physical functioning, a culprit in the attenuation of support in the context of cancer, and critical outcomes such as general quality of life, social functioning and emotional distress. These results support the utility of the SRCE-I as a mediating mechanism in this Italian sample of women with breast cancer.

With the limitation that these analyses were conducted with a cross-sectional dataset, the results do suggest that in a sequence of events starting with the physical limitations imposed by cancer and its treatments and ending with an outcome related to quality of life, social functioning and emotional well-being, SRCE may mitigate social losses and perhaps even enhance existing relationships. The mediation results also portend a role for SRCE in interventions in the lives of those who are at risk for social support loss or who have experienced distress due to the shrinking of the quantity or quality of their social network.

The cross-cultural replication of the psychometric findings as well as the mediation analyses may be accounted for by the theoretical basis for SRCE. That is, as opposed to restricting the person with cancer to the role of “support recipient”, SRCE, which is based on Self-regulation [30] and Self-efficacy Theories [28], promotes personal agency and activation [51]. Thus, persons with cancer may be thought of as agents in the construction, maintenance, or enhancement of their social environment. Thus, the core SRCE construct appears to be relevant in the diverse, albeit Western, cultural settings in which it has been tested thus far; however, the exact enactment of this process of maintaining or enhancing social support may vary to some extent even in those different cultural settings.

Along those lines, according to Optimal Matching Theory [24], social support is maximized when the need for support and the provision of support are aligned [22]. Alternatively, when need and provision of support are not aligned, social support may be not only not beneficial but harmful by perhaps promoting “sick role” behavior as opposed to promoting recovery [23]. Thus, SRCE may be a model for helping patients to understand the interpersonal dynamics of social support and advocate for their need for support as well as to be able to help others understand that mismatched support provision may not be helpful. That is, SRCE can be a platform for placing social support in the context of patients’ close interpersonal relationships and encouraging them to develop constructive conversations that support the alignment of need and provision.

### Limitations

Although the current study did replicate prior work on the social relationship coping efficacy scale [25, 31], the sample was limited to women who were receiving treatment for breast cancer in northern Italy. Therefore, the results are limited in generalizability and would need to be replicated on men and other-gendered cancer patients and in more diverse geographical locations in Italy and elsewhere. In addition, the mediation analyses were useful in illuminating the role of SRCE, however, those analyses were conducted on a cross-sectional sample, not in a longitudinal design, which would be more definitive because causal arguments can be forwarded regarding the relationships between variables.

### Clinical implications

The psychometric data and mediation model-testing of the Italian version of the SRCE scale affirm that social support and close supportive relationships are linked to positive outcomes. Thus, as in the US and Greek versions of the SRCE scale, in this Italian sample of breast cancer patients, self-efficacy for maintaining and enhancing social relationships may be critical for mitigating loss of social support and promoting quality relationships that can help adaptation along the breast cancer care journey. Consequently, the SRCE-I can represent a screening instrument to identify those patients who are at risk for a reduction in the quantity or quality of social relationships and, thus also at risk for an increase in loneliness or social isolation. In line with that use, the SRCE-I can also function as the basis for developing specific psychosocial interventions aimed at improving social support for patients with cancer.

## Supplementary Information

**Additional file 1:** Regression analyses and English version of the Social Relationship Coping Efficacy Scale.

## Data Availability

The datasets generated and/or analyzed during the current study are not publicly available while the manuscript is under review, but are available from the corresponding author on reasonable request. The dataset and supplemental materials have been archived in CurateND (Link. 10.7274/2514nk3512z).
