# Supplementary material for: Assessment of socio-relational self-efficacy in breast cancer patients: Italian validation of the social relationship coping efficacy scale (SRCE-I)
Source: BMC Psychol. 2022 Nov 3;10:248. doi: 10.1186/s40359-022-00966-7 (PMC9632030; doi:10.1186/s40359-022-00966-7)
Supplement: Supplementary file 1 — Additional file 1: Regression analyses and English version of the Social Relationship Coping Efficacy Scale. [file 40359_2022_966_MOESM1_ESM.docx]

**Supplemental Materials**

**Appendix A**

**Impact of demographic and medical variables on SRCE-I: simple and multiple regression analysis results**

| **Variable** | **Simple regression models (N=181)** | | | **Multiple regression model (N=130)** | | |
| --- | --- | --- | --- | --- | --- | --- |
|  | **R-square** | **beta** | **p-value** | **R-square** | **beta** | **p-value** |
| Age | .054** | -0.23** | .002 | .243* | -.34** | .003 |
| Marital status (married vs other) | .008 | .09 | .233 |  | .13 | .178 |
| Educational level (high education vs low education) | .004 | .06 | .397 |  | -.03 | .770 |
| Employment Status (worker vs other) | .001 | -.02 | .753 |  | -.11 | .253 |
| Spirituality (practicing religion vs other) | .000 | .01 | .885 |  | .01 | .953 |
| Network (yes vs no) | .006 | -.08 | .309 |  | -.05 | .579 |
| Hobbies (yes vs no) | .002 | -.05 | .545 |  | -.15~ | .094 |
| Volunteering (yes vs no) | .014 | .12 | .107 |  | .09 | .300 |
| Time from diagnosis | .000 | -.02 | .824 |  | .04 | .650 |
| Performance status ECOG | .023* | -0.15* | .041 |  | -.11 | .251 |
| Tumor stage [N = 146, not staged was excluded] | .013 | .11 | .170 |  | .10 | .261 |
| Metastasis (yes vs no) [N = 179] | .006 | -.08 | .289 |  | -.15 | .141 |
| Toxicity (3-4 vs 1-2) [N = 150] | .011 | .11 | .194 |  | -.01 | .905 |
| Previous surgery (yes vs no) [N = 179] | .000 | .02 | .802 |  | -.01 | .943 |
| Previous radiotherapy (yes vs no) [N = 179] | .007 | -.08 | .261 |  | .01 | .922 |
| Previous chemotherapy (yes vs no) [N = 180] | .041** | 0.20** | .006 |  | .19~ | .090 |
| Hormone treatment (yes vs no) [N = 179] | .005 | -.07 | .369 |  | -.11 | .213 |

**Appendix B**

**Social Relationship Coping Efficacy Scale**

**English Version**

This survey contains many things that a person might do during and after cancer treatment. We are interested in how confident you are that you can do those things. Be sure your ratings are about your confidence even if you have not done it in the past. So, your ratings are about your confidence that you can do these things now or in the near future.

Please read each item. Then rate that item on how confident you are that you can do that behavior. Circle a number on the scale. If you circle a “9” you are totally confident that you can do that behavior. If you circle a “1” you are not at all confident that you can do that behavior. Numbers in the middle mean that you are somewhat confident that you can do that behavior. Be sure your ratings reflect your confidence even if you have not done it in the past.

Please rate all items. If you are not sure about an item please rate it as best you can.

1. **Doing my part to**  NOT AT ALL MODERATELY TOTALLY

**maintain close**  CONFIDENT CONFIDENT CONFIDENT

**relationships** 1 2 3 4 5 6 7 8 9

2. **Managing stress in**  NOT AT ALL MODERATELY TOTALLY

**my relationships** CONFIDENT CONFIDENT CONFIDENT

1 2 3 4 5 6 7 8 9

3. **Asking for help**  NOT AT ALL MODERATELY TOTALLY

**when I need it**  CONFIDENT CONFIDENT CONFIDENT

1 2 3 4 5 6 7 8 9

4. **Seeking emotional**  NOT AT ALL MODERATELY TOTALLY

**support from others**  CONFIDENT CONFIDENT CONFIDENT

1 2 3 4 5 6 7 8 9

5. **Coping with stress**  NOT AT ALL MODERATELY TOTALLY

**in my close**  CONFIDENT CONFIDENT CONFIDENT

**relationships** 1 2 3 4 5 6 7 8 9

6. **Doing my part to**  NOT AT ALL MODERATELY TOTALLY

**help family members** CONFIDENT CONFIDENT CONFIDENT

**accept/understand** 1 2 3 4 5 6 7 8 9

**my diagnosis**

7. **Doing my part to**  NOT AT ALL MODERATELY TOTALLY

**help my friends** CONFIDENT CONFIDENT CONFIDENT

**accept/understand my** 1 2 3 4 5 6 7 8 9

**diagnosis**

8. **Adjusting to the ways** NOT AT ALL MODERATELY TOTALLY

**cancer affects my**  CONFIDENT CONFIDENT CONFIDENT

**family** 1 2 3 4 5 6 7 8 9

9. **Coping with the ways** NOT AT ALL MODERATELY TOTALLY

**that cancer affects**  CONFIDENT CONFIDENT CONFIDENT

**my personal** 1 2 3 4 5 6 7 8 9

**relationships**

10. **Managing conflict**  NOT AT ALL MODERATELY TOTALLY

**with those closest**  CONFIDENT CONFIDENT CONFIDENT

**to me** 1 2 3 4 5 6 7 8 9
